# Voluntary running rescues the defective hippocampal neurogenesis and behaviour observed in lipocalin 2-null mice

**DOI:** 10.1038/s41598-018-38140-y

**Published:** 2019-02-07

**Authors:** Ana Catarina Ferreira, Ashley Novais, Nuno Sousa, João Carlos Sousa, Fernanda Marques

**Affiliations:** 1 Life and Health Sciences Research Institute (ICVS), School of Medicine, University of Minho, Campus Gualtar, Braga, Portugal; 2 ICVS/3B’s - PT Government Associate Laboratory, Braga/Guimaraes, Portugal

## Abstract

The continuous generation of new neurons in the adult mammalian hippocampus is a form of neural plasticity that modulates learning and memory functions, and also emotion (anxiety and depression). Among the factors known to modulate adult hippocampal neurogenesis and brain function, lipocalin-2 (LCN2) was recently described as a key regulator of neural stem cells (NSCs) proliferation and commitment, with impact on several dimensions of behaviour. Herein, we evaluated whether voluntary running, a well-known regulator of cell genesis, rescue the deficient adult hippocampal neurogenesis observed in mice lacking LCN2. We observed that running, by counteracting oxidative stress in NSCs, reverses LCN2-null mice defective hippocampal neurogenesis, as it promotes NSCs cell cycle progression and maturation, resulting in a partial reduction in anxiety and improved contextual behaviour. Together, these findings demonstrate that running is a positive modulator of adult hippocampal neurogenesis and behaviour in mice lacking LCN2, by impacting on the antioxidant kinetics of NSCs.

## Introduction

In the adult mammalian brain, the continuous generation of new neurons in the subgranular zone (SGZ) of the dentate gyrus (DG) in the hippocampus contributes to modulate local neural plasticity and network dynamics^1^. The potential significance of this remodeling process has been shown to directly impact learning and memory^2^, contextual fear conditioning^3^, synaptic plasticity^1^ and brain function, both in health and disease^4^. In fact, impaired or deficient neurogenesis in the hippocampus has been associated with the development of several neuropsychiatric disorders, including dementia^5,6^, anxiety^7^ and depression^8^. Noticeably, over the past years, numerous studies have identified both intrinsic and environmental factors that regulate adult neurogenesis and, hence, modulate behaviour via neurogenic-dependent mechanisms. Among them, lipocalin-2 (LCN2) was recently reported as a key regulator in adult neurogenesis orchestration^9^ and animal behaviour^10^. Primarily reported as an acute-phase protein in the innate immune response^11^, LCN2 recently emerged as an important modulator of brain physiology and disease^12^, required for the maintenance of hippocampal integrity, plasticity and function^10,13^. Specifically, studies with a knockout mouse model for *Lcn2* (LCN2-null) revealed that LCN2 is involved in emotional and cognitive behaviours^10^, in the control of neuronal excitability and dendritic remodelling^10,13^, and in the generation of new cells^9^.

Of notice, in the last few years, running has received particular attention due to its potential in improving cognitive functions^14,15^ and emotional behaviours^16^, as it promotes synaptic plasticity^17^ and hippocampal neurogenesis^18^. Particularly, voluntary running was shown to robustly increase SGZ neural progenitors proliferation^18,19^, the generation of new neurons^18^, and to enhance spatial pattern separation^14^ and contextual discrimination^20^. In fact, exercise-induced neurogenesis is currently explored as a strategy to overcome and rescue the decrease in neurogenesis and memory associated with pathology^21^ and aging^22^.

Here, we investigated whether voluntary running could revert the impairments in the process of hippocampal neurogenesis observed in the absence of LCN2. We describe that exercise improves LCN2-null mice impaired NSCs proliferation and survival, as it specifically rescued the redox status of NSCs and allowed the cell cycle progression of neural progenitors. This, in turn, increased the generation of newborn neurons, and contributed to partially reduce anxiety and improve contextual discrimination in LCN2-null mice.

## Results

### Voluntary running increases cell proliferation and survival

**Figure 1:**
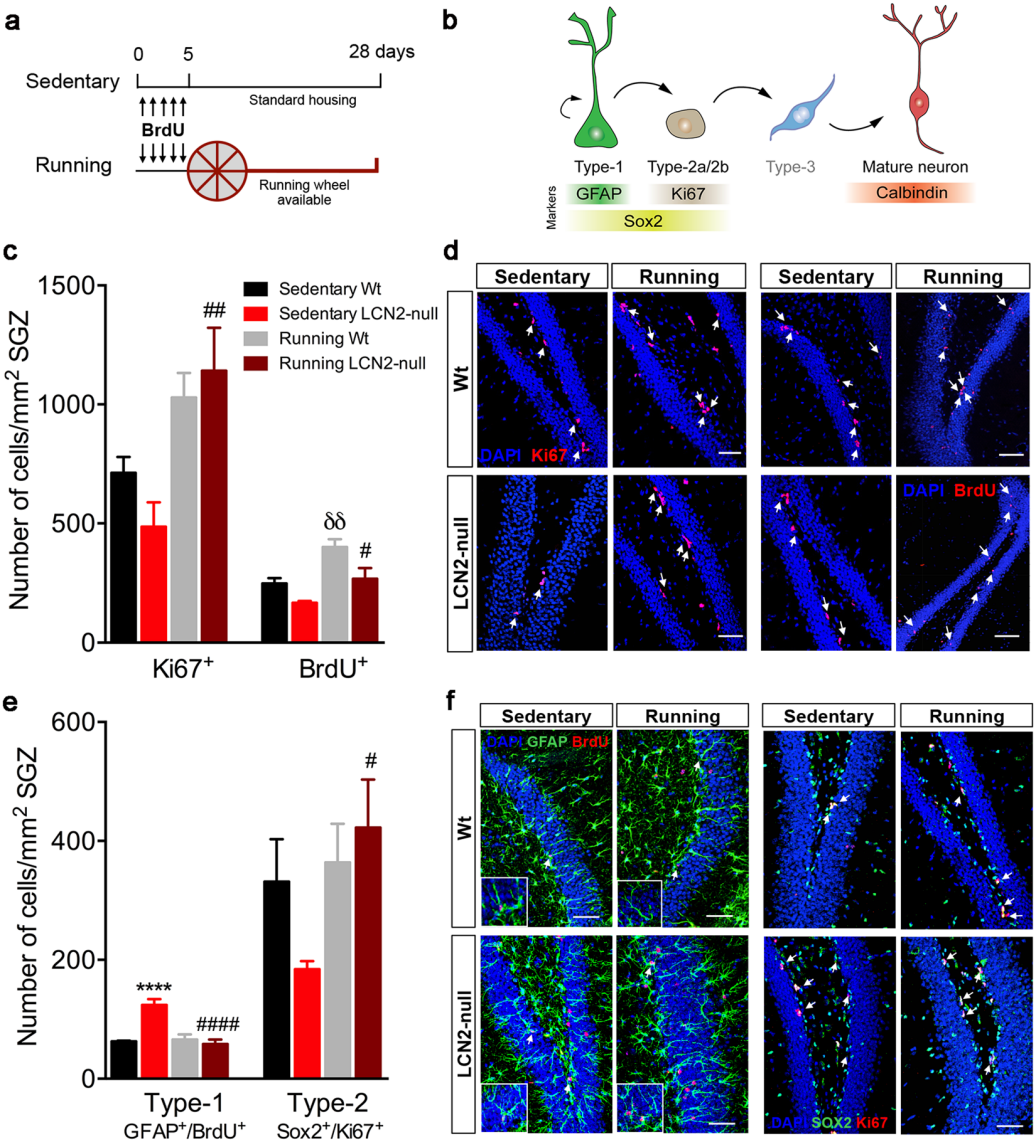
Voluntary running promotes hippocampal cell proliferation and survival, potentiating the transition of quiescent to proliferating NSCs in LCN2-null mice. (**a**) Schematic diagram of the experimental paradigm of voluntary running and of the BrdU injection protocol used. For 28 days, Wt and LCN2-null mice were assigned as running, i.e. animals housed to have free access to a running wheel, and as sedentary, housed under standard housing conditions with no running wheel available. (**b**) Representative illustration of the hippocampal neurogenic process, including cellular types and the specific markers used. (**c**) Running increased cell proliferation (Ki67^+^ cells) in LCN2-null mice, and cell survival (BrdU^+^ cells) in both Wt and LCN2-null mice (n = 4–6 per group). (**d**) Representative confocal images of Ki67 and BrdU immunostaining (indicated by white arrows) in the SGZ of the DG of Wt and LCN2-null sedentary and running mice. (**e**) Quantitative analysis of radial quiescent type-1 (GFAP^+^/BrdU^+^) and amplifying type-2 stem cells (Sox2^+^/Ki67^+^) after 28 days of running revealed a significant decrease in type-1 population in LCN2-null mice, and a consequent increase in type-2 cells (n = 5 mice per group). (**f**) Representative confocal images of GFAP^+^/BrdU^+^ and Sox2^+^/Ki67^+^ immunostaining in the SGZ of sedentary and running mice of both genotypes (indicated by white arrows). Scale bars, 50 μm. Data from the sedentary animals is the same as described in Ferreira *et al*.^9^, and are presented as mean ± SEM and were analyzed by two-way ANOVA with Bonferroni’s multiple comparison test. *Denotes differences between sedentary Wt and LCN2-null mice; ^δ^between sedentary and running Wt; ^#^between sedentary and running LCN2-null mice. ^#^*p* ≤ 0.05, ^δδ,##^*p* ≤ 0.01, ****^,####^*p* ≤ 0.0001.

Firstly, cell proliferation in the hippocampus of running Wt and LCN2-null mice was compared to that of sedentary animals published in Ferreira *et al*.^9^. As shown in that publication, the number of Ki67^+^ proliferating cells was lower in sedentary LCN2-null mice, when compared to sedentary Wt (Fig. 1c,d)^9^. Four weeks of voluntary exercise (Fig. 1a) robustly increased the number of Ki67^+^ cells in the SGZ (running effect: F_1,16_ = 16.3, *p* = 0.001), specifically promoting a significant increase in cell proliferation at the hippocampus of LCN2-null mice (3-fold increase, *p* = 0.003 *versus* sedentary LCN2-null mice; Fig. 1c,d). In addition, analysis of the total number of BrdU^+^ cells, as a measure of cell survival in the SGZ, revealed that exercise significantly increased this population (running effect: F_1,14_ = 19.9, *p* = 0.0005), both in Wt (*p* = 0.007) and LCN2-null SGZ (*p* = 0.03), compared to the respective sedentary genotypes (Fig. 1c,d).

### LCN2-null mice hippocampal quiescent NSCs respond to voluntary running, thus promoting progenitors proliferation

We next sought to ascertain whether the observed increased cell proliferation and survival were related to regulatory events in the pool of stem cells. Since we used a label-retaining BrdU protocol at the beginning of the voluntary running protocol (Fig. 1a), this allowed us to label the quiescent pool of cells and, 28 days later, analyze both the progeny that exited the cell cycle and differentiated, and the pool of cells that retained BrdU and remained quiescent. Quantitative analysis of quiescent type-1 stem cells (Fig. 1b) that present a radial glia-like morphology and co-express BrdU and GFAP^23^ revealed that, after running, while no effect was observed in Wt mice, a significant reduction in the number of radial type-1 stem cells was observed in LCN2-null mice (*p* < 0.0001), to numbers similar to those observed in sedentary Wt mice, as published in Ferreira *et al*.^9^ (Fig. 1e,f). Of notice, this was similarly observed when we analyzed type-1 nonradial stem cells in the DG, identified as Sox2^+^ Ki67^−^ cells and compared to the sedentary LCN2-null mice published in Ferreira *et al*.^9^ (*p* = 0.07; Supplementary Figure S1a). In addition, analysis of amplifying type-2 progenitors, identified as Sox2^+^ Ki67^+^ cells (Fig. 1b), showed that running was effective (running effect: F_1,16_ = 4.6, *p* = 0.04) in promoting the transition of type-1 to type-2 stem cells and, specifically in LCN2-null mice, it significantly increased the number of type-2 stem cells (*p* = 0.03 *versus* sedentary LCN2-null animals; Fig. 1e,f). In fact, voluntary exercise tends to increase the percentage of Sox2^+^ cells in cycle in the SGZ of LCN2-null mice (7% increase, *p* = 0.08 *versus* sedentary LCN2-null animals; Supplementary Figure S1b).

**Figure 2:**
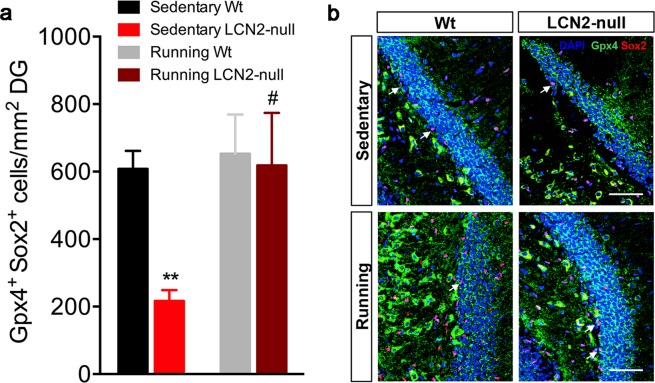
Voluntary running improves the expression of an antioxidant enzyme in LCN2-null NSCs. (**a**) Cellular quantification of the number of Sox2^+^ NSCs that co-express the antioxidant enzyme Gpx4 showed a significant improved antioxidant regulation in running LCN2-null mice (n = 5 mice per group). (**b**) Representative confocal images of Sox2 and Gpx4 co-labeling at the DG (scale bar, 50 μm). Data from the sedentary animals is the same as described in Ferreira *et al*.^9^, and are presented as mean ± SEM and were analyzed by two-way ANOVA with Bonferroni’s multiple comparison test. *Denotes differences between sedentary Wt and LCN2-null mice; ^#^between sedentary and running LCN2-null mice. ^#^p ≤ 0.05, **p ≤ 0.01.

Interestingly, we have previously described that LCN2-null mice lack a proper antioxidant regulation in NSCs, which contributed to an impaired cell cycle regulation and deficits in the generation of new cells^9^. As we observed that running improved cell proliferation and promoted the cell cycle progression of NSCs, we next analyzed its effects on the expression levels of the antioxidant enzyme glutathione peroxidase 4 (Gpx4) in Sox2^+^ cells. Quantification of Gpx4^+^ Sox2^+^ cells revealed a significant increase in LCN2-null mice DG after running (*p* = 0.01 *versus* sedentary LCN2-null; Fig. 2a,b). Yet, four weeks of running did not exert any effect in Wt animals.

### Exercise rescues LCN2-null mice behavioral deficits and increases the number of newborn mature neurons

**Figure 3:**
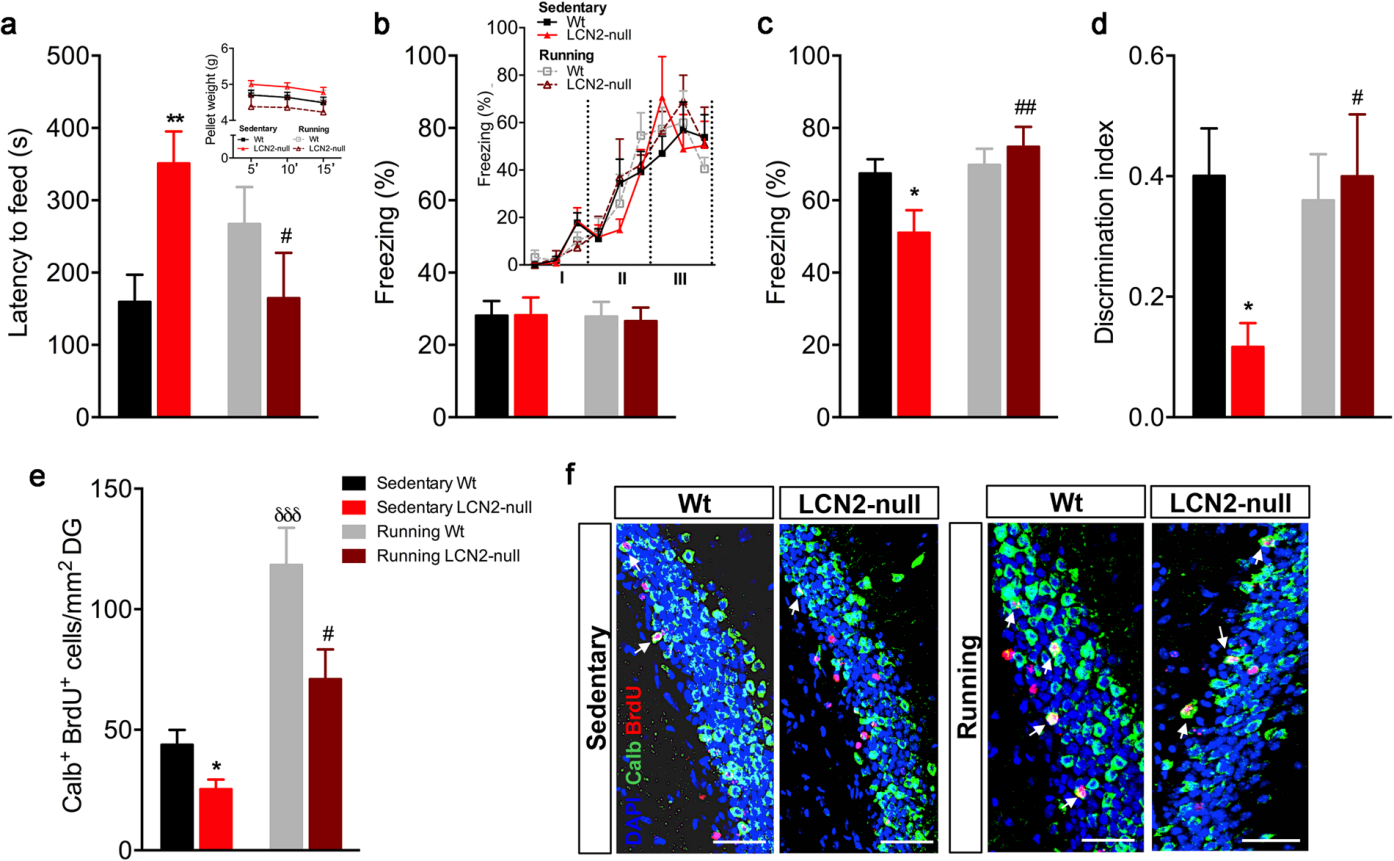
Exercise reduces anxiety and improves contextual discrimination in LCN2-null mice, by increasing the generation of newborn mature neurons. (**a**) Anxiety assessment in the novelty-suppressed feeding paradigm showed that sedentary LCN2-null mice take longer time to feed, which is decreased after voluntary running (n = 6–10 mice per group). No differences between groups in appetite drive were observed. (**b**) Freezing behaviour during the training session, represented as the total percentage of freezing and as the progressive increased freezing acquired along the trials of light/shock presentation (Block I-III representing the three light-shock pairings), was similar between groups (n = 6–10 mice per group). (**c**) Impaired contextual retrieval in context A by sedentary LCN2-null mice is rescued by exercise. (**d**) Discrimination index affected by the absence of LCN2 is re-established after housing LCN2-null mice with running wheels. (**e**) Quantitative analysis of the number of newborn mature neurons at DG revealed that exercise promoted a significant increase in both Wt and LCN2-null mice (n = 5 mice per group). (**f**) Representative images of Calbindin (Calb) and BrdU immunostaining at DG (indicated by white arrows). Scale bar, 100 μm. Data from the sedentary animals is the same as described in Ferreira *et al*.^9^, and are presented as mean ± SEM and were analyzed by two-way ANOVA with Bonferroni’s multiple comparison test. *Denotes differences between sedentary Wt and LCN2-null mice; ^δ^between sedentary and running Wt; ^#^between sedentary and running LCN2-null mice. *^,#^*p* ≤ 0.05, **^,##^*p* ≤ 0.01, ^δδδ^*p* ≤ 0.001.

Since voluntary running effectively rescued LCN2-null impaired cell cycle regulation of NSCs in the SGZ, we next analyzed the effect of exercise on behaviour and the generation of new neurons in the absence of LCN2. We firstly addressed the effect of exercise in the novelty-suppressed feeding (NSF) test, a paradigm that assesses anxiety-like behaviour. In line with our previous results^10^, we observed that sedentary LCN2-null mice display an anxious-like phenotype, as they presented a significant increased latency to feed (*p* = 0.008; Fig. 3a). Of interest, this phenotype was rescued by voluntary running, since LCN2-null mice decreased the latency of time required to feed after exercise (*p* = 0.02 *versus* sedentary null mice; Fig. 3a). Importantly, running had no effect on this behavioral dimension in Wt mice (Fig. 3a). No differences were observed between experimental groups in the appetite drive (Fig. 3a). In the EPM test, only a partial reversion of the anxious phenotype was observed in the LCN2-null mice after running, without reaching statistical significance (Supplementary Figure S2), although it induced an anxious phenotype in Wt animals (Supplementary Figure S2).

Next, we tested the animals for their ability to discriminate between overlapping contextual presentations after the voluntary running protocol. Herein, we added to the published work^9^ that, while no differences between groups after running were observed during training (similar learning curve of freezing time along the trials of shock exposure; Fig. 3b), running led to an overall significant improvement in contextual discrimination by LCN2-null mice (running effect: F_1,20_ = 6.07, *p* = 0.02). We observed that the results obtained in Ferreira *et al*.^9^ regarding the impaired contextual retrieval (*p* = 0.05) and discrimination index (*p* = 0.01) presented by sedentary LCN2-null mice were rescued by exercise. The percentage of freezing upon re-exposure to context A (*p* = 0.009; Fig. 3c), and the animals’ capacity to discriminate between the two different contexts (*p* = 0.02; Fig. 3d) was promoted by voluntary running, but only in animals lacking LCN2.

We next assessed the generation of newborn mature neurons in the hippocampus. For that purpose, we identified the progeny of cells that was labeled in the beginning of the protocol (Fig. 1a) and that exited the cycle and differentiated into mature neurons (identified as Calb^+^ BrdU^+^ cells). Quantification of this population showed that exercise had a strong impact in the generation of new neurons (running effect: F_1,17_ = 30.4, *p* < 0.0001), and that it promoted a significant increase of Calb^+^ BrdU^+^ cells in the DG of LCN2-null mice (3-fold increase, *p* = 0.01 *versus* sedentary null mice; Fig. 3e,f). Particularly for this cell population, housing with running wheels also promoted a substantial increment in Wt animals (3-fold increase, *p* = 0.0004) when compared to the sedentary controls published before^9^ (Fig. 3e,f).

Moreover, a correlation analysis revealed an association between the number of newborn neurons (Calb^+^ BrdU^+^ cells) and the behavioral performance of LCN2-null mice in the NSF and CFC tests (Supplementary Table 1). This analysis shows that the increase in the number of newborn neurons observed in the DG of LCN2-null mice, after exercise, may contribute to the improvements in anxiety, at least in the NSF (*p* = 0.07), and in contextual retrieval at the CFC test (*p* = 0.06; Supplementary Table 1), similarly to what others have reported on the correlation between the number of cells in the DG and the performance during acquisition in the water maze^24^.

Together, our data reveals that voluntary exercise partially rescues LCN2-null mice impaired anxiety but it completely reverted contextual discriminatory behavior, possibly through its pro-neurogenic effects. Voluntary exercise effectively sustained an increase in cell proliferation and survival in LCN2-null mice, providing long-lasting increases of proliferation, cell survival and production of newborn neurons.

## Discussion

**Figure 4:**
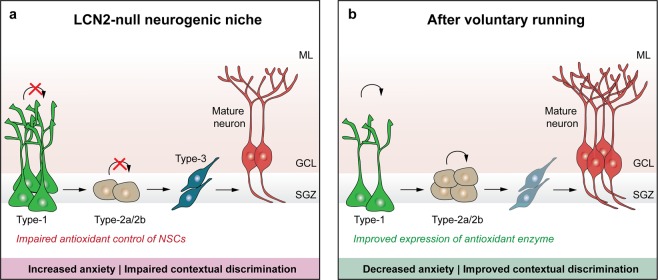
Schematic diagram of the effects of voluntary running in LCN2-null mice hippocampal neurogenesis and behaviour. In LCN2-null DG, voluntary running stimulates the transition of type-1 to type-2 stem cells, increasing cell proliferation and survival, and the generation of newborn mature neurons. These positive effects in the neurogenic process translated into decreased anxiety and improved contextual discriminative behaviours. GCL, granule cell layer; ML, molecular layer; SGZ, subgranular zone.

The relevance of LCN2 in the control of adult hippocampal neurogenesis has just recently emerged in the current literature^9^. With well-known roles in cell physiology in other systems^25^, the lack of LCN2 was recently described to induce deficits in NSCs proliferation and commitment, with impact on hippocampal-dependent contextual fear discrimination^9^ and anxiety^10^. Of interest, LCN2-null impaired neurogenesis resulted from alterations in endogenous oxidative stress mechanisms of NSCs, cell cycle arrest and death, an effect that is iron-mediated since it was restored by the addition of an iron chelator^9^. Herein, we showed that voluntary running triggered a pro-neurogenic effect in the mouse model of LCN2-null-impaired cell genesis. Specifically, we found that running is sufficient to overcome the deficits in cell proliferation and differentiation observed in the absence of LCN2, which was accompanied by a partial improved anxiety and reversion of contextual discriminative behaviours (Fig. 4).

Throughout the recent years, the pro-neurogenic benefits of exercise have been widely described, with reports showing enhanced adult hippocampal cell proliferation and neurogenesis^18,26^, and improvements in specific hippocampal-dependent tasks, such as pattern separation^14^ and contextual fear conditioning^20^. In fact, exercise-induced neurogenesis is quite often used as a strategy to overcome and rescue deficits in neurogenesis and memory-associated impairments^22^. Notably, housing of Wt and LCN2-null mice with running wheels for 28 days effectively improved LCN2-null mice neurogenic and behavioral deficits. Specifically, we observed that exercise was able to recruit LCN2-null quiescent NSCs into cell cycle, promoting the transition of cells from a quiescent to a proliferative state, a process that was impaired in LCN2-null mice^9^. Moreover, we found that accompanying all this process, running restored the antioxidant response of NSCs in LCN2-null mice, which we found to be impaired in the absence of LCN2 under physiological conditions^9^. This is in accordance with previous reports that showed voluntary running to preferentially recruit quiescent cells for cell division^27^, thus promoting the proliferation of type-2 cells, as well as their survival and maturation within the hippocampus^28^. Also, the pro-proliferative cellular effects have been shown to rely on the NSCs cell cycle dynamics. For instance, some authors have shown that running induces quiescent NSCs to enter into the cell cycle^29^, while it promotes cell cycle exit of fast progenitor cells^27^. It is possible that NSCs from LCN2-null mice, although highly quiescent, present a proliferative potential that is higher than those of Wt mice that can be recruited to increase the neurogenic process in response to external stimulations, such as running. In fact, as a consequence of a decrease in the pool of stem cells, we observed that voluntary running increased the percentage of type-2 progenitor cells present in the active phase of the cycle in the SGZ of LCN2-null mice.

A multitude of intrinsic factors, triggered in response to exercise, are considered to underline the mechanisms of exercise-induced hippocampal neurogenesis. Particularly, vasculature-associated trophic factors, including insulin and vascular-endothelial growth factors, have been implicated as mediators of running-induced neurogenesis, since both are increased in running animals (both serum and hippocampal levels)^30,31^. In the case of LCN2-null mice, at this point it is not clear through which mechanisms voluntary running is promoting improvements in neurogenesis and behaviour. Of notice, we have previously observed that LCN2-null NSCs present a deficient antioxidant regulation that lead to increased oxidative stress, cell cycle arrest and death^9^. Interestingly, voluntary running improved the antioxidant regulation of LCN2-null NSCs, as quantified by the number of Sox2^+^ cells that express the antioxidant enzyme Gpx4. This, at least in part, could explain the observed improvements in cell cycle progression of NSCs in LCN2-null mice, thus impacting on their survival. In fact, this is in line with other reports showing that regular exercise improves cognitive function and decreases oxidative damage^31,32^. Nevertheless, further analyses should evaluate if this effect on Gpx4-expressing cells is extended to other stages of the hippocampal neurogenesis.

The effectiveness of the voluntary running protocol applied was evident by the significant increase in the generation of newborn neurons that it promoted in the DG of both Wt and LCN2-null mice. Importantly, this increase in neurogenesis potentially provided the plasticity required to partially reduce anxiety and restore contextual fear discrimination in LCN2-null mice. In accordance, several authors have shown that voluntary running, by modulating neurogenesis^33^, has anxiolytic effects^16,33^. Contrastingly, the increased generation of newborn neurons in Wt animals, promoted by running, did not render better performances in the CFC task. Similarly, others have shown that running-increased neurogenesis does not potentiate contextual fear memory^34^. Moreover, and partially in accordance with reports describing that voluntary running induces anxiety-like behaviour in the open field and light/dark box tests, by increasing hippocampal neurogenesis^35^, herein Wt animals, although presenting a normal phenotype when tested for anxiety-like behavior in the NSF, presented an anxious phenotype in the EPM.

Taken together, our results suggest that voluntary running is pivotal in the control of neurogenesis and that impaired cell genesis, due to the lack of LCN2, is highly responsive to this external modulation. Voluntary running was able to revert the hippocampal neurogenesis dependent behavioral deficits presented by LCN2-null mice.

### Concluding remarks

This work provides evidence on the effects of hippocampal neurogenesis modulation in a mouse model of LCN2-null-impaired cell genesis. The capacity to modulate neurogenesis and, in the case of voluntary running, to overcome behavioral deficits displayed by LCN2-null mice, reinforces the importance of LCN2 as a key player in the regulation of neural plasticity, for proper brain functioning.

## Material and Methods

### Animal Experiments

Experiments were conducted in 10-weeks-old male mice lacking *Lcn2* (LCN2-null), and their respective wild-type (Wt) littermate controls, in a C57BL/6J mice background. Animals were obtained from crossing heterozygous animals, and mice genotype was confirmed by polymerase chain reaction. Mice were housed and maintained accordingly with the guidelines for the care and handling of laboratory animals in the Directive 2010/63/EU of the European Parliament and of the Council, in a controlled environment at 22–24 °C and 55% humidity, on 12 h light/dark cycles and fed with regular rodent’s chow and tap water *ad libitum*. All animal procedures were conducted in accordance with the approved guidelines and regulations on animal care and experimentation stated in the EU directive 2010/63/EU and were approved by the Portuguese national authority for animal experimentation, *Direção Geral de Veterinária* (DGAV; ID: DGAV9457).

#### Voluntary running

Wt and LCN2-null mice were randomly housed under standard housing conditions (Sedentary group), or with free access to a running wheel (Running group) for 28 days^18^. To analyze the effects of exercise on the progeny of NSCs and progenitors survival, animals were intraperitoneally injected with 50 mg/kg of BrdU (Sigma Aldrich, St. Louis, MO, USA) twice a day for 5 consecutive days in the beginning of the running protocol (Fig. 1a). Behavioral tests were conducted at the end of the voluntary running protocol, next described in detail.

To note, the group of sedentary animals herein reported are the same as previously described in Ferreira *et al*.^9^, since the voluntary running group was performed simultaneously as the sedentary group already published.

### Behaviour

#### Novelty suppressed-feeding

To examine anxiety-like behaviour in response to a novel environment, animals were assessed in the novelty-suppressed feeding (NSF) paradigm. After 18 h of food-deprivation, animals were placed in an open field arena (Med Associates Inc., St. Albans, VT, USA), where a single food pellet was placed in the center. The latency of time to feed was recorded and used as a measure of anxiety-like behaviour. After reaching the pellet, animals were returned to the home cage where they were allowed to eat pre-weighted food for 5, 15 and 30 min as a measure of appetite drive.

#### Elevated plus maze

Anxiety-like behavior was assessed through the elevated plus maze (EPM) test consisting of two opposite brightly illuminated open arms (51 cm × 10 cm) and two opposite dark closed arms (50.8 cm × 10.2 cm × 40.6 cm) elevated 72.4 cm above the floor (ENV-560; Med Associates Inc.). Mice were placed individually in the center of the maze and allowed to freely explore it for 5 min. The percentage of time spent in the open arms was used as an index of anxiety-like behavior.

#### Contextual fear conditioning

Contextual fear conditioning (CFC) was conducted in ventilated sound-attenuated chambers with internal dimensions of 20 cm wide × 16 cm deep × 20.5 cm high (SR-LAB, San Diego Instruments, San Diego, CA, USA), with a light mounted directly above the chamber to provide illumination. The floor consisted of a stainless grid that was attached to a shock generator (Coulbourn Instruments, Allentown, PA, USA) for foot shock delivery. A fan mounted on one side of each box provided ventilation and background noise, which was only set on upon context changing. Mice behaviour was recorded by digital video cameras mounted above the conditioning chamber, and freezing behaviour was manually scored by a blind observer using the Etholog V2.2 software^36^. Freezing was defined as the complete absence of motion for a minimum of 1 sec.

The fear conditioning procedure was conducted for 2 days. On day 1, mice were placed in the conditioning chamber and received pairings between light and a termination shock (1 sec, 0.5 mA), spared from each other with an interval of 20 sec. Mice received three light-shock pairings with an inter-trial interval of 2 min between each block, and the first light presentation started 3 min after the mouse was placed into the chamber. Animals returned to their home cage 30 sec after the last shock was presented. The chambers were cleaned with 10% ethanol between each mouse. On the following day, mice were tested for conditioned fear to the training context and placed in the same chamber (context A), where the training contextual conditions remained from the previous day, but with no presentation of the conditioned stimulus. Mice were placed into the chambers for 3 min and the entire session was scored for freezing. Two hours after this, animals were presented to a novel context (context B), where the grid was removed and black plastic inserts covered the floor and walls of the chamber. Also, the chamber was scented with a paper towel dabbed with vanilla extract, placed underneath the chamber floor and the ventilation fan was set on. In addition, the experimenter wore a different style of gloves and changed the lab coat. Chambers were cleaned with 10% ethanol between runs and mice were kept in a different holding room before testing. Each mouse was placed into the chamber for 3 min and freezing was scored for the entire session. Parameters analyzed included the percentage of time freezing during the training session, the total percentage of time freezing in the context (A) and (B), and the index of discrimination between contexts as the ratio of percentage of time freezing (contexts A − B)/percentage of time freezing (contexts A + B).

### Tissue preparation and immunohistochemistry

At the end of the behavioral assessment, mice were anesthetized with an intraperitoneal injection of a mixture of ketamine hydrochloride (150 mg/kg, Imalgene 1000) plus medetomidine (0.3 mg/kg, Dorben), and transcardially perfused with 0.9% saline, followed by perfusion with cold 4% paraformaldehyde (PFA) solution. Brains were removed, postfixed for 1 h in 4% PFA, cryoprotected in 30% sucrose overnight, and then embedded in Optimal cutting temperature compound (ThermoFisher Scientific, Waltham, MA, USA), snap-frozen and kept frozen at −20 °C until further sectioning. Posterior serial coronal sections (20 μm) were cut in a cryostat and collected to slides for immunohistochemistry. For BrdU immunostaining, antigen retrieval by heat with 10 mM citrate buffer (Sigma) was performed on the fixed tissue sections, followed by DNA denaturation with HCl (Sigma) for 30 min at room temperature (RT). An additional blocking step with 10% normal fetal bovine serum (FBS; Invitrogen, Carlsbad, CA, USA) in a solution of PBS 0.3% Triton X-100 (PBS-T; Sigma) was also performed for 30 min at RT. Primary antibodies incubation, diluted in blocking solution, occurred overnight at RT as following: rabbit anti-glial fibrillary acidic protein (GFAP, 1:200; DAKO, Glostrup, Denmark), rabbit anti-calbindin (1:300; Abcam, Cambridge, UK), rat anti-BrdU (1:100; Abcam), mouse anti-Sox2 (1:200; Abcam), rabbit anti-Ki67 (1:300; Millipore, Billerica, MA, USA) and rabbit anti-glutathione peroxidase 4 (Gpx4, 1:50; Abcam). Fluorescent secondary antibodies (Invitrogen) anti-rat, anti-rabbit and anti-mouse, combined to Alexa 594 or to Alexa 488, were used to detect the respective primary antibodies at a dilution of 1:500 (in PBS-T) for 2 h at RT. To stain the nucleus, sections were then incubated with 4′,6-diamidino-2-phenylindole (DAPI, 1:1000; Sigma), after which slides were mounted with Immu-Mount (ThermoFisher).

### Confocal imaging and quantitative analysis

Fluorescence images of the DG were acquired using a Olympus FV1000 confocal microscope (Olympus, Hamburg, Germany). Fields were acquired using Z-scan with a step of 1 μm between each confocal plane. All sections prepared for comparison were analyzed at the same time, using the same acquisition parameters. The quantification rates of the parameters analyzed were estimated by the analysis of 3–4 sections per animal. The number of double positive cells at the SGZ of the DG was calculated using the Olympus Fluoview software (Olympus), and normalized for the respective area (mm^2^).

### Statistical analysis

All experiments were performed and analyzed by the same experimenter, blind to the animals’ genotype or group treatment under assessment. Variables followed a Gaussian distribution as revealed by the D’Agostino & Pearson normality test. Data are reported as mean ± standard error (S.E.M.). The number of biological replicates (n) is specified in the legend of each figure. Statistical significant differences between groups were determined using two-way ANOVA, followed by Bonferroni’s multiple comparison test. Values were considered statistically significant for *p* ≤ 0.05 (*, # or δ), *p* ≤ 0.01 (**, ## or δδ), *p* ≤ 0.001(***, ### or δδδ) and *p* ≤ 0.0001(****, #### or δδδδ).

## Supplementary information

Supplementary data
