# Supplementary material for: Voluntary running rescues the defective hippocampal neurogenesis and behaviour observed in lipocalin 2-null mice
Source: Sci Rep. 2019 Feb 7;9:1649. doi: 10.1038/s41598-018-38140-y (PMC6367505; doi:10.1038/s41598-018-38140-y)
Supplement: Supplementary file 1 — Supplementary data [file 41598_2018_38140_MOESM1_ESM.docx]

**Supplementary information**

##### **Voluntary running rescues the defective hippocampal neurogenesis and behaviour observed in lipocalin 2-null mice**

Ana Catarina Ferreira^1,2^, Ashley Novais^1,2^, Nuno Sousa^1,2^, João Carlos Sousa^1,2^, Fernanda Marques^1,2^

**Supplementary figures**

**Figure S1**

**
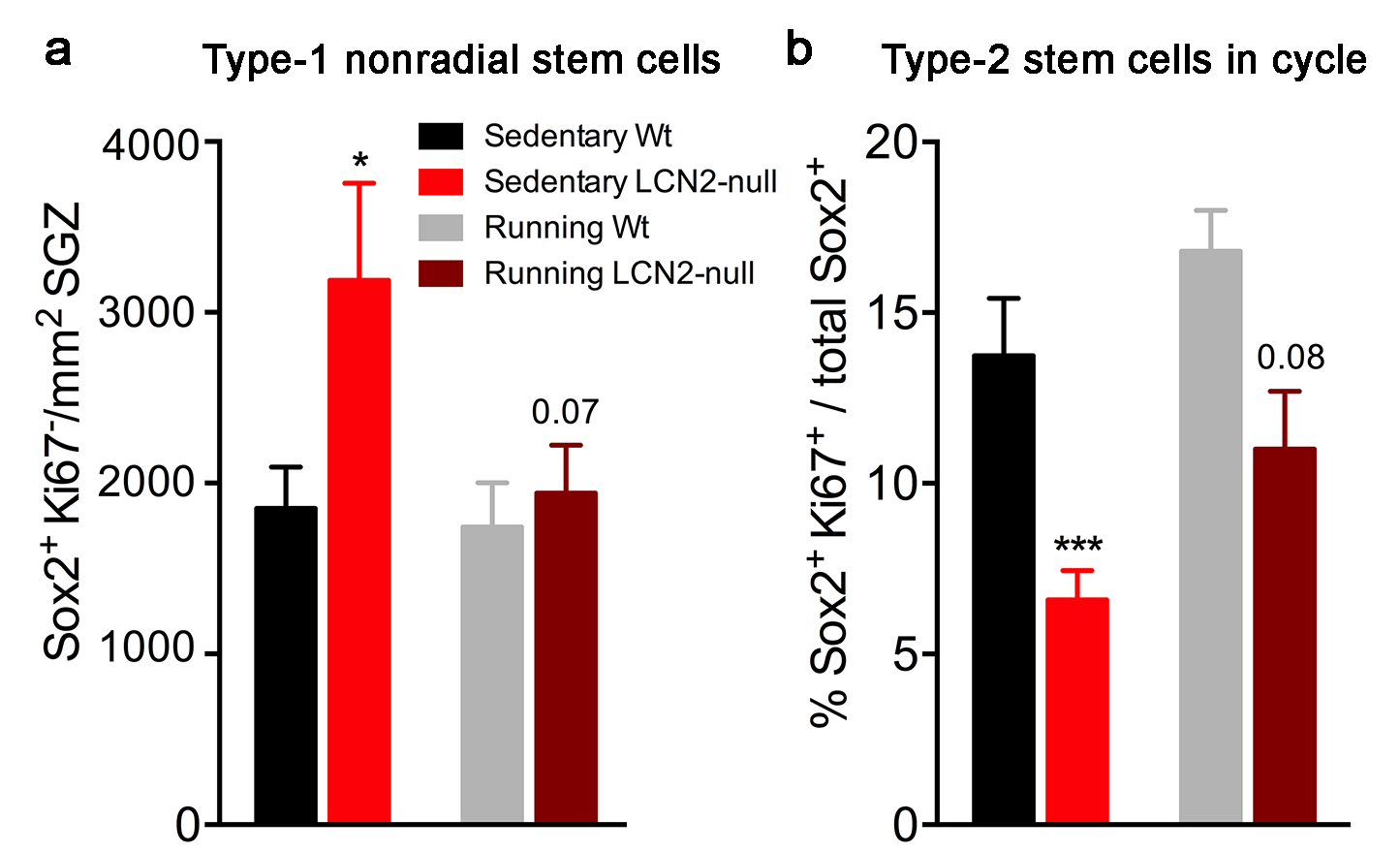
**

**Figure S2**

**
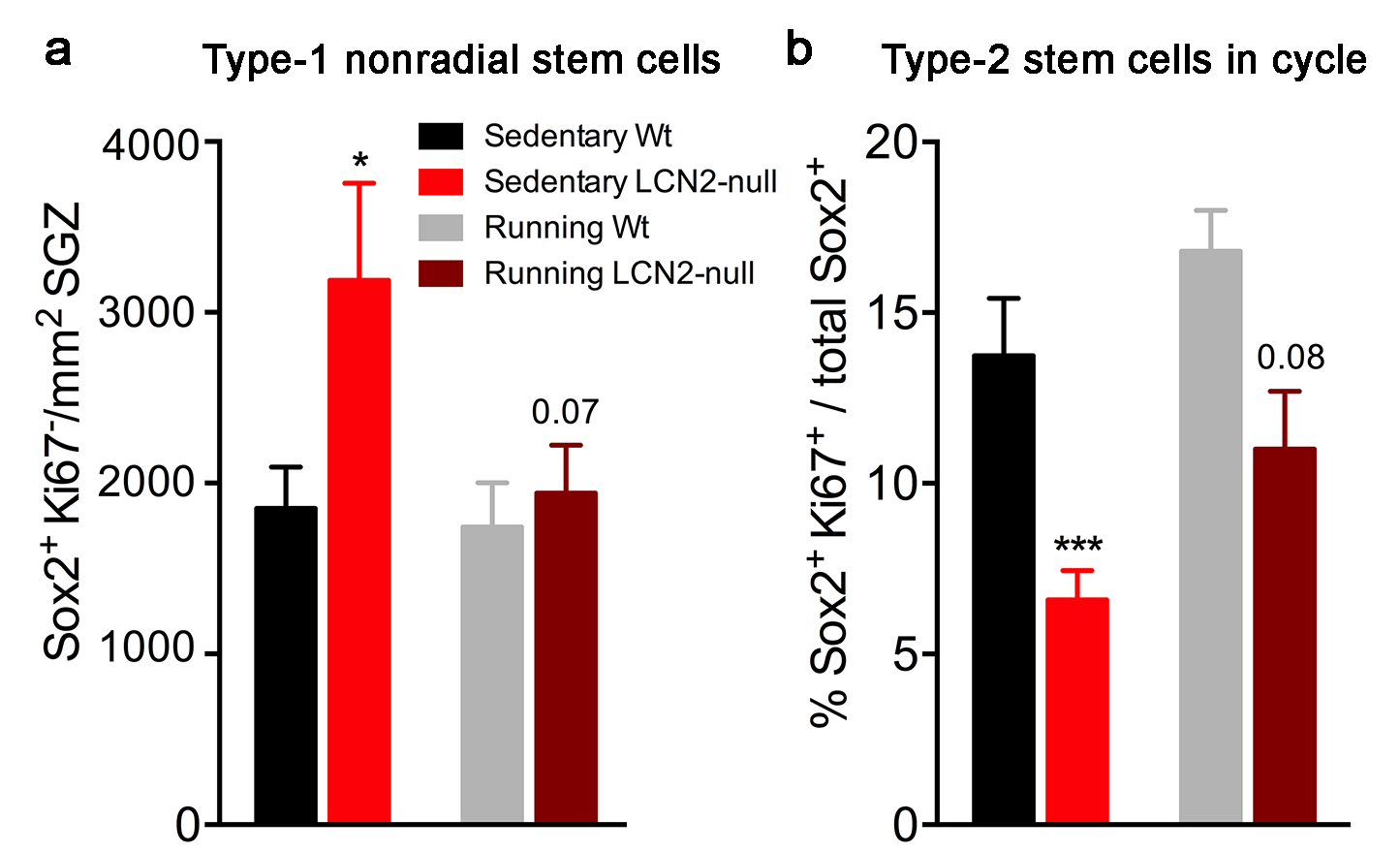
**

**Table S1**

| Newborn neurons  Calb^+^/BrdU^+^ | NSF  Latency to feed (s) | | CFC  Freezing (%) | |
| --- | --- | --- | --- | --- |
|  | Wt | LCN2-null | Wt | LCN2-null |
| p value | 0.26 | 0.07 | 0.15 | 0.06 |
| *r* | 0.5455 | -0.8373 | 0.7484 | 0.9322 |

**Figures legends**

**Figure S1:** Running promotes the transition of quiescent to proliferating neural stem cells in LCN2-null mice. (**a**) Quantification of nonradial Ki67^-^ Sox2^+^ type-1 stem cells after exercise revealed a decrease in LCN2-null mice. (**b**) Analysis of the proportion of type-2 stem cells in cycle showed that exercise promotes the regulation of cell cycle in the absence of LCN2. Data are presented as mean ± SEM and were analyzed by two-way ANOVA with Bonferroni’s multiple comparison test. *Denotes differences between sedentary Wt and LCN2-null mice. *p≤0.05, ***p≤0.001.

**Figure S2:** Anxiety-like behavior assessed in the EPM test. Analysis of the time spent in the open arms in the EPM showed no significant effect of running in LCN2-null mice, while inducing an anxious phenotype in Wt animals. Data are presented as mean ± SEM and were analyzed by two-way ANOVA with Bonferroni’s multiple comparison test. *Denotes differences between sedentary Wt and LCN2-null mice; ^δ^between sedentary and running Wt. *,^δ^p≤0.05.

**Table S1:** Correlations between neurogenesis and behavioral performances in the NSF and CFC, after voluntary running.
